# A rapid ethnographic study of breastfeeding in the North and South of Italy

**DOI:** 10.1186/1746-4358-1-14

**Published:** 2006-09-05

**Authors:** Sofia Quintero Romero, Rosa Bernal, Chiara Barbiero, Raquel Passamonte, Adriano Cattaneo

**Affiliations:** 1 Centro per la Salute del Bambino, Trieste, Italy; 2 Clacyd, Córdoba, Argentina; 3 Unit for Health Services Research and International Health, Institute of Child Health IRCCS Burlo Garofolo, Via dei Burlo 1, 34123 Trieste, Italy

## Abstract

**Background:**

The past ten years have witnessed a rising trend in the prevalence and duration of breastfeeding in Italy, but breastfeeding rates increase in an unequal way; they are higher in the North of Italy than in the South. The purpose of this study was to describe the experiences, expectations and beliefs of a sample of mothers, and to identify differences, if any, between the North and the South of Italy.

**Methods:**

The study was conducted in two regions of Italy, Friuli Venezia Giulia in the Northeast and Basilicata in the South. Two hundred and seventy-nine mothers of infants and children 6 to 23 months of age were interviewed using an 85-item questionnaire including closed and open questions on infant feeding experiences and beliefs, sources of information and support, reasons for intended and actual choices and practices, and some demographic and social variables. Face-to-face interviews were conducted between May 2001 and September 2002. Quantitative and qualitative methods were used for data analysis.

**Results:**

The distribution of the mothers by age, education, employment and parity did not differ from that of the general population of the two regions. The reported rates of initiation and duration of breastfeeding were also similar: 95% started breastfeeding, exclusive breastfeeding was 32% at three and 9% at six months, with 64% and 35% of any breastfeeding, respectively. Some differences were reported in the rates of full breastfeeding, reflecting different ages of introduction of non-nutritive fluids. These, as well as nutritive fluids – including infant formula – and complementary foods, were introduced far too early. Advice on infant feeding was generally provided by health professionals and often was not based on up-to-date recommendations. Mothers were generally aware of the advantages of breastfeeding, but at the same time reported problems that they were not able to solve alone or through social and health system support. Most mothers would welcome the support of a peer counsellor. More mothers in Basilicata than in Friuli Venezia Giulia reported difficulties with breastfeeding related to returning to work and were not familiar with their rights on breastfeeding and maternity leave.

**Conclusion:**

Programmes for the protection, promotion and support of breastfeeding in these and similar regions of Italy should concentrate on better training of health professionals with regards to lactation management, communication, and counselling skills. The addition of trained peer counsellors could reinforce the work done by the health system and, through community involvement, could help change social prejudice in the mid- and long-term. The differences between regions should be taken into account in formulating these programmes to avoid increasing, and possibly to decrease, the current gaps.

## Background

The past ten years have witnessed a rising trend in the prevalence and duration of breastfeeding in Italy [1,2]. Between 1995 and 1999 the initiation rate has gone up from 85% to 91%, while the rates of any breastfeeding at six and twelve months have increased from 19% to 47% and from 4% to 12%, respectively. Exclusive breastfeeding rates, however, lag behind what is recommended at international and national levels [3-5]. Moreover, they do not seem to increase at the same pace: in 1999, only 5% of infants were exclusively breastfed at six months, with no improvement since 1995 [2].

Most importantly, breastfeeding rates increase in an unequal way; they are higher among mothers with higher levels of income and education [6,7], which is consistent with reports from other countries in Europe and elsewhere [8-12]. They are also higher in the North of Italy than in the South. In the northeastern region of Friuli Venezia Giulia (FVG), full (exclusive plus predominant) [13] breastfeeding was 88% at discharge and 41% at four months in 1999 [1]; in the South of Italy full breastfeeding was 79% at discharge and about 30% at four months [14]. The level of development of health and welfare services, and of access to them, may not fully explain these disparities. Other personal and social determinants have been found to be associated with the prevalence and duration of breastfeeding [15,16], and it has been recently suggested that individual attitudes related to the intended duration of breastfeeding or the mother's perception of her partner's preference might be important predictors of infant feeding choices and practices [17,18].

The purpose of this rapid ethnographic study was to describe the experiences, expectations and beliefs of a sample of mothers, and to identify differences, if any, between the North and the South of Italy. In addition, the study was intended to offer some insight into the local determinants of breastfeeding to regional programmes for the promotion of breastfeeding planning to train peer counsellors, as previously done in Mexico [19].

## Methods

The study was conducted in two regions of Italy, FVG in the Northeast and Basilicata (BAS) in the South. Eight health districts, four in each region, were selected to represent urban (Trieste, Matera), rural (Monfalcone and Cervignano, Tricarico and Maratea) and mountain (Tolmezzo, Chiaromonte) areas. The National Health System lists of people registered as residents in each district were used to identify all the mothers of at least one infant or child 6 to 23 months of age (index infant and child). Simple random sampling, with probability proportional to the size of each district, was then used to locate the mothers to be enrolled in the study. It was estimated that 133 participants in each region were needed to identify, with 95% confidence and 80% power, an odds ratio (OR) of 0.5 between FVG and BAS if 25% of mothers in one region intended to breastfeed exclusively their children up to four months of age. The sample was increased to 150 per region to account for invalid data. Exclusive and full breastfeeding were defined as recommended by WHO [13].

Before enrolment started, an 85-item questionnaire including closed and open questions on infant feeding experiences and beliefs, sources of information and support, reasons for intended and actual choices and practices, and some demographic and social variables, was developed and tested. At enrolment, each mother was first contacted by telephone to assess her willingness to participate and to make an appointment in an agreed location: at home, in a nearby clinic, in the research office, or in any other place that the mother considered convenient. An informed consent was read and signed at the beginning of each 60- to 90-minute face-to-face interview carried out by either SQR or RB. If a mother included in the sample declined to participate, she was immediately replaced by the next one in the district register, following alphabetical order.

Quantitative data were directly entered in EpiInfo files. Qualitative data were first categorised by two members of the research team (CB, AC) and, after sorting out any disagreement, entered in the EpiInfo files. Statistical analysis was carried out by CB, RP and AC using SPSS 9.0 software. Chi-square tests, t-test and one-way ANOVA were used to identify statistically significant differences between regions. The study protocol was approved by the ethics committee of the Institute of Child Health IRCCS Burlo Garofolo in Trieste.

## Results

Between May 2001 and September 2002, a total of 279 mothers accepted to participate, 143 in FVG (Trieste 50, Monfalcone 24, Cervignano 18, Tolmezzo 51) and 136 in BAS (Matera 61, Tricarico 16, Maratea 48, Chiaromonte 11). The distribution of these mothers by age, education, employment and parity did not differ from that of the general population, thus confirming the validity of the simple random sampling procedure. The mean age was 33 years in FVG and 31 in BAS (range 15–44 in both). One hundred and forty women (50%) had completed secondary school and 57 (20%) had a university degree. There were significant differences as far as employment is concerned: 49 (36%) were unemployed in BAS compared with 39 (27%) in FVG (p = 0.007), with a lower proportion of women employed as clerks and professionals in the former. Seventy-eight mothers (57%) were multiparae in BAS compared with 63 (44%) in FVG (p < 0.05); 31 mothers (11%) had three or more children; 95% of those with a previous child had breastfed.

### Infant feeding practices

The reported rate of initiation of breastfeeding was 95% (266/279). Reported exclusive breastfeeding was 44% at one, 32% at three, and 9% at six months of age. The reported rate of any breastfeeding was 86% at one, 64% at three, 35% at six, and 5% at twelve months of age. In all these cases there were no significant differences between FVG and BAS. There were differences (p < 0.05), however, in the reported rates of full breastfeeding: 71% vs 75% at one, 61% vs 66% at three, and 22% vs 16% at six months of age in FVG and BAS, respectively.

**Figure 1:**
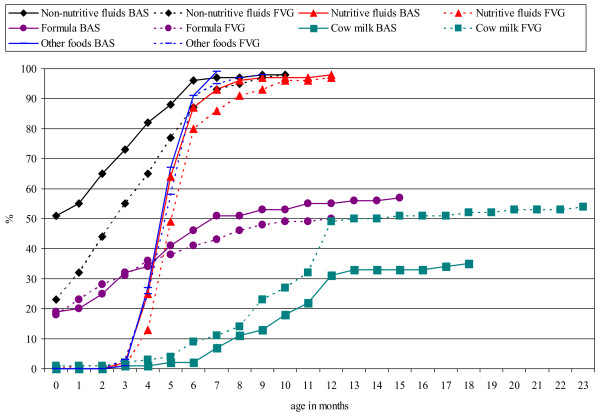
Introduction of different foods and fluids by age and region (cumulative percentage).

Figure 1 shows the cumulative percentage by age and region of index infants and children given foods and fluids other than breastmilk. Non-nutritive fluids, mainly sweetened or unsweetened water, but also different kind of teas, were given earlier in BAS (51% in the first month) than in FVG (23%). Nutritive fluids such as fruit juices and, less frequently, broth and soup, were introduced between three and four months, slightly later in FVG than in BAS. The pattern of introduction of infant formula was similar in both regions; 56 index infants (20%) had it from the first month, reaching a plateau around 50% from age 7–9 months. About 50% of index infants were not given infant formula, either because they were breastfed or because cow milk was introduced from six months onwards. Finally, other foods, both commercial and home made, were introduced at four months and given to virtually all index infants by seven months of age, with no difference between FVG and BAS.

### Advice on infant feeding

One hundred and forty-eight mothers (53%) mentioned health professionals as their first source of advice, in FVG the family paediatrician (43%), assigned by the National Health System to every newborn, and the hospital or community midwife (55%), in BAS the family paediatrician (66%) and lower levels of health professionals (23%), while the midwife was almost never mentioned (3%). Among those who received health professional advice, 128 (86%) stated that they had followed it. Grandmothers, more often than friends and members of the family, were mentioned as another source of advice; only 44% (FVG) and 53% (BAS) of mothers declared that they had followed this kind of advice. The media were mentioned as the last source of advice, mainly specialised magazines and books on a 1:1 and 10:1 ratio in FVG and BAS, respectively. As far as the type of advice is concerned, 24 mothers (9%) reported that a health professional (a paediatrician in 20 cases) had told them to stop breastfeeding (when the baby was less than a month old in 10 cases). In 58 cases (21%) the advice to stop breastfeeding was given by other family members (29), friends (13) and grandmothers (11); only three fathers gave this advice. Fifty-one mothers in FVG (36%) and 67 (49%) in BAS reported the use of infant formula based on health professional advice; in more than 90% of these cases the advice had come from the family paediatrician, in 34% the baby was less than a month old, in 29% between one and three months, in 17% between four and six months. The same advice came also from other family members (36%), grandmothers (30%), friends (16%) and fathers (14%). The introduction of other foods was almost always recommended by health professionals, mostly the family paediatrician, at about four (25% of cases), five (39%) and six (27%) months of age.

### Feeding choices

Mothers were asked to rank the factors influencing their feeding choices from a list of seven (Table 1). "Optimal nutrition and growth" was ranked first by 218 mothers (78%), while "makes me feel free" was never ranked first or second and more often ranked in 6^th ^and 5^th ^place; "keeps me slim" was the factor ranked last more often (51%). Mothers were then given a list of foods and fluids and were asked to check whether they would have given them to their babies less than six months old in a range of circumstances (Table 2). Most mothers would have given breast milk in most situations, but a number would not have done so if the infant had diarrhoea or colic. In addition, they would have given some foods and fluids improperly, such as water and camomile at birth. None of them considered breast milk as the only food needed in the first six months, even in case of illness. There were some statistically significant differences between FVG and BAS: in the latter, fewer mothers would have given breast milk in case of diarrhoea and colic, and infant formula in all circumstances; but more mothers would have given water, camomile, fruit juice, rice water, vegetable soup and cereal porridge in many circumstances.

**Table 1:** Rank order assigned by mothers to seven different factors influencing their feeding decisions.

**Factor influencing infant feeding decisions**	**Percentage of mothers ranking each factor as:**						
	
	**1**^**st**^	**2**^**nd**^	**3**^**rd**^	**4**^**th**^	**5**^**th**^	**6**^**th**^	**7**^**th**^
Provides optimal nutrition and growth	78	12	5	2	0	1	1
It is clean and sterile	10	22	27	16	14	9	3
Babies are healthier	9	53	19	11	5	2	1
It is easy and convenient	2	11	31	35	14	5	2
It helps keeping me slim and beautiful	2	1	5	7	12	24	51
It is cheap	0	2	6	15	24	22	31
It makes me feel free	0	0	7	15	32	36	10

**Table 2:** Foods and fluids that mothers (%) would give their babies in different circumstances, by region.

**Foods and fluids**	**At birth**		**Diarrhoea**		**Thirst**		**Colic**		**Fever**	
	
	**FVG**	**BAS**	**FVG**	**BAS**	**FVG**	**BAS**	**FVG**	**BAS**	**FVG**	**BAS**
Breast milk	99	100	94*	77*	98	93	91*	77*	97	93
Infant formula	55*	41*	50*	26*	58*	38*	52*	27*	56*	38*
Cow milk	6	4	4	4	9	4	4	2	9	4
Water	24*	42*	39*	58*	73	78	30	41	57*	74*
Camomile	19*	32*	32	26	49	53	45*	73*	43	50
Fruit juice	0	0	1	1	16*	33*	0	2	8*	19*
Rice water	1	3	8*	36*	2*	8*	1*	15*	3	7
Vegetable soup	0	0	7	11	18	18	8	10	15*	28*
Cereal porridge	0	0	2*	15*	8*	16*	4*	13*	8*	21*
Homogenised baby foods	0	0	7	10	14	9	6	8	13	18

* p < 0.05

### Reasons to breastfeed

Mothers were asked to name up to three reasons to breastfeed. Out of 771 answers, 28% were related to protecting infant health, 26% to providing optimal nutrition, 19% to improved attachment, 10% to practical convenience, 4% to cost, 1% to maternal health, the remaining 12% to a series of more generic considerations. Mothers also named 430 favourite aspects of breastfeeding; they gave prominence to attachment (56%) and practical convenience (17%). A single question probed the optimal duration of exclusive breastfeeding: 118 mothers (42%) stated that it should last six months or more, 50 (18%) five, 35 (13%) four, 19 (7%) three, 9 (3%) two, 9 (3%) one and 29 (10%) less than one; 10 mothers (4%) did not answer the question.

Asked about problems they had had with breastfeeding in the first six months, 92 mothers (33%) reported that they had had no problem. Table 3 shows the distribution of the 371 answers given by the remaining 187 mothers. Mothers named also 299 unpleasant aspects of breastfeeding, 40% related to the heavy commitment it requires (time, responsibility, sacrifice, lack of freedom) and 30% to other negative experiences (sore nipples, engorgement, concern about milk supply). Cultural factors such as the fear of spoiling the breast, the prediction of a less independent child, and the concern about breastfeeding in public, were also mentioned (16%), as well as stress and fatigue (8%) and other myths and beliefs (5%). Among the 504 reasons to stop breastfeeding, 37% were related to the inadequacy of breast milk for the growing needs of the child, 30% to maternal factors (fatigue, stress, disease, medicines, new pregnancy), 15% to return to work, 12% to the fear of inadequate psychological development associated with prolonged breastfeeding ("the breast had become a vice, not a source of nutrients"), 3% to breast problems (mastitis), and the remaining 3% to miscellaneous reasons.

**Table 3:** Proportional distribution of 371 infant feeding problems in the first six months, reported by 187 mothers.

**Category of problem**	**Example**	**N.**	**%**
Breast	Sore nipple, engorgement, mastitis	86	23
Milk	Insufficient, inadequate	82	22
Baby	Colic, constipation, excess crying	42	11
Growth	Growth less than expected	42	11
Mother	Tired, bored, unwilling to continue	32	9
Latch	Difficult latching, too short/long feeds	23	6
Pumping	Hand, mechanical, electric	16	4
Maternal illness	Depression, drugs	15	4
Work	At home, at workplace	14	4
Infant illness	Fever, immaturity	9	2
Other		10	3
Total		371	99

### Reasons to feed infant formula

The 493 reasons to give infant formula were similarly distributed: inadequacy of breast milk (54%), maternal factors (16%), return to work (12%), breast problems (5%) and miscellaneous factors (8%); 14 mothers (5%), however, would give their babies infant formula because they consider it a high-quality food. Among the 213 positive aspects of feeding infant formula, 24% were related to cleanliness and safety, 21% to practical convenience (including the fact that someone else, usually the father, could feed the baby), 16% to the enrichment in micronutrients, 13% to the possibility to verify the amount eaten, 12% to the fact that it is the only alternative for mothers who cannot breastfeed; the remaining positive aspects were less specific and included overcoming the stigma associated with breastfeeding in public.

Among the 327 negative aspects; 30% were related to practical inconveniences (the need to dilute, sterilise, store, re-warm, wake up at night), 22% to nutritional inadequacy when compared with breast milk, 12% to the difficulties it creates for attachment and bonding, 12% to cost, 7% to bad taste, 17% to other miscellaneous features of infant formula. Asked about the optimal age for the introduction of infant formula, 107 mothers in FVG (75%) compared with 41 in BAS (30%) answered that ideally they would never give it to their babies; the remaining answers were distributed between one and twelve months of age, skewed towards birth in BAS more than in FVG.

### Other infant feeding topics

Table 4 shows the proportion of mothers who expressed their degree of disagreement or agreement, on a scale of 0 to 9, in response to 10 statements regarding infants less than six months. The reactions to statements 1, 5, 6, 8 and 10 may be considered a sign of adequate knowledge, but the irregular distribution of agreement/disagreement on the remaining statements reveals a lack of information. Mothers were uncertain about the influence of their diets on breast milk and the protection breastfeeding provides against infectious diseases (statements 2 and 3), split up on the reason why doctors prescribe infant formula and on the need to give water to exclusively breastfed infants (statements 4 and 9), and expressed a 30% disagreement on the proportion of mothers who do not have enough milk (statement 7).

**Table 4:** Proportion (%) of mothers who disagree or agree, on a scale of 0 to 9, in response to 10 statements regarding infants less than six months.

**Statements:**	**Maximum disagreement ← → Maximum agreement**										
	
	**0**	**1**	**2**	**3**	**4**	**5**	**6**	**7**	**8**	**9**	DK*
1. Infants grow better if breast milk is complemented with infant formula	78	4	2	2	4	3	1	1	0	4	0
2. The milk of a mother who does not eat well does not meet the infant's needs	18	4	9	5	11	10	4	8	4	26	2
3. An exclusively breastfed infant is less susceptible to diarrhoea and ear infection	18	7	5	4	6	6	4	5	7	32	6
4. When the doctor prescribes infant formula supplements, breast milk is insufficient	49	5	2	2	2	5	6	4	5	19	0
5. If a breastfeed lasts more than 10 minutes, nipples become sore	70	5	4	3	3	2	0	3	2	7	2
6. After a C-section, mothers have to wait at least 24 hours before they start breastfeeding	63	2	2	1	2	2	1	2	2	10	14
7. Only a small proportion of mothers, less than 1%, does not have enough milk	12	3	7	3	6	7	3	8	5	36	11
8. There is no need whatsoever to give foods and fluids other than breast milk	13	3	2	3	5	6	4	4	8	52	0
9. Water should be given to all exclusively breastfed infants, especially in hot weather	31	5	6	4	5	7	4	6	6	26	0
10. Sweet water or infant formula should be given to all newborns while waiting for the onset of milk production	59	6	4	2	3	3	2	4	1	14	3

* DK = don't know

Two hundred and thirty-nine mothers (86%) would welcome the support offered by a trained peer counsellor; 130 (54%) would prefer such skilled support to the one of a health professional, 41 (17%) would consider it equivalent, 80 (33%) would prefer the latter. One hundred and thirty-eight mothers (49%) considered that a personal successful experience is essential to provide meaningful support; 59 (21%) stated that they would feel at ease with a peer more than with a professional.

Finally, the association between breastfeeding and employment was investigated. Of the 152 mothers continuing to breastfeed, 33/66 (50%) had to leave their job in BAS compared with 13/86 (15%) in FVG (p < 0.05). In this region, the median maternity leave was nine months, in BAS it was only five months. Asked about their rights, 220 mothers (79%) could name at least six among the following: maternity, parental and sick infant leave; the right to a part time job and to breastfeeding breaks; the norms that regulate night shifts and dangerous tasks; the rights to a salary and of not being dismissed. However, when more details were asked, many mothers had inadequate knowledge of some details, e.g. length of the maternity, parental and sick child leave; age of the child that entitles the mother to a part time job and exemption from night shifts and dangerous tasks; number and duration of breastfeeding breaks; percentage of salary cut at different post-partum periods. More importantly, significant differences emerged between FVG and BAS on some of these rights (Table 5). For example, mothers in FVG were almost twice as likely to know the exact duration of maternity and parental leave, while 55% of mothers in BAS, compared with 33% in FVG, could not recall the number, the length and the duration of the allowed breastfeeding breaks.

**Table 5:** Proportion (%) of mothers with varying levels of detailed knowledge of rights related to maternity leave and breastfeeding, by region.*

Knowledge of details**	**Maternity leave**		**Parental leave**		**Sick child leave**		**Breastfeeding breaks**	
	
	**FVG**	**BAS**	**FVG**	**BAS**	**FVG**	**BAS**	**FVG**	**BAS**
Good	87	48	77	38	17	28	21	13
Fair	8	22	17	26	71	23	46	32
Poor	5	30	6	36	13	50	33	55

* all differences between regions significant at p < 0.05 level.

** good = all details; fair = some details; poor = none of the details.

## Discussion

To our knowledge, this is the first modern investigation of the infant feeding beliefs, attitudes, experiences and expectations of mothers in Italy, and certainly the first comparing a northern to a southern region. In 2000, Elizabeth D. Whitaker published a long historic and ethnographic study on breastfeeding in Italy, looking at its evolution from the 19^th ^century onwards and discussing specific beliefs she had come across while investigating in the Emilia Romagna region [20]. She has shown the contrast between instinctual or intuitive maternal knowledge and "common sense", and the high degree of complexity and precision in standards for infant feeding and growth generated by state and medical control, first in the fascist period and then in the post 2^nd ^World War years. She states that "it is not surprising that most women give up breastfeeding rather soon after starting, given the complicated technique they are expected to follow", and concludes that "as compliance with medical management has increased, this outcome has been ensured given the conflict between regimented norms for breastfeeding and the psychobiology of lactation." [[20], p237]. She is referring mainly to test weighing and rigid scheduled feeding. Her book, however, though extremely interesting for a cultural debate, does not offer practical hints to professionals engaged in programmes for the promotion of breastfeeding [20].

We think that our study may help fill this gap. Our results show that, with the exception of different opinions on duration of exclusive breastfeeding, knowledge of the importance of breast milk and breastfeeding at different ages and in different situations may be considered as adequate. Practices, however, fall short of standard recommendations, as shown by the reported rates of breastfeeding. These should be interpreted with caution, because of the long recall period we used, as opposed to the standard 24-hour recall recommended by WHO [13]. But even with caution, it appears that the rates of exclusive breastfeeding in the first six months are low. Other foods and fluids, especially non-nutritive fluids, are introduced too early, more in BAS than in FVG. This is reflected in the higher percentage of mothers in the former region that would give water, camomile, fruit juice, rice water, vegetable soup and cereal porridge in different circumstances. Hence the need for breastfeeding programmes that concentrate more on individual and community support for exclusive breastfeeding than just on promotion of breastfeeding.

The practical advice given by health professionals, the first source reported by mothers, has to be brought into line with WHO and other professional recommendations. Midwives should be encouraged and supported to play their important role, especially in BAS. Our study shows that more than 50% of the reported problems could be attributed to inadequate feeding technique or lactation management that could be overcome if skilled health professionals and/or peer counsellors were available. It also shows that paediatricians still provide incorrect advice, e.g. when they put limits on the duration of a feed, and often recommend infant formula supplements when what is probably needed is reassurance on the adequacy of breast milk, careful assessment of positioning and latching to prevent or relieve sore nipples, advice for the identification of and response to feeding cues, guidance on baby-led duration of feeds, and practical support for the management of other problems interfering with optimal lactation. Special support should be provided to mothers following caesarean section in areas, such as the South of Italy, where the rate of this mode of birth is very high [21].

At a community level, more has to be done, especially in the South of Italy, to empower mothers vis-à-vis their breastfeeding rights and to protect them from advertisements of breast milk substitutes conveyed by popular parents' magazines [22]. There are obviously problems that originate from mothers. Many of them, for example, believe that nipple fissures occur more often in women with light skin and red hair; some even think that there is a causal association between these factors, and therefore may not seek the support of a skilled professional or peer counsellor to manage nipple fissures. Others consider that their perceived lack of breast milk is related to their diet or to a panic episode. A breastfeeding programme focussed on identified priorities and run by well trained professionals will not eradicate these beliefs easily; mid- and long-term cultural changes are needed to replace myths and modify attitudes.

## Conclusion

To conclude, we think that based on our findings, programmes for the protection, promotion and support of breastfeeding in these and other regions of Italy should concentrate on better training of health professionals with regards to lactation management, communication, and counselling skills. The addition of trained peer counsellors could reinforce the work done by the health system and, through community involvement, could help change social prejudice in the mid- and long term. The final aim should be the empowerment of mothers through improved knowledge on how to breastfeed, for a free and informed choice. The minor differences between regions revealed by this study do not imply differences in the overall strategic approach. We think that these differences are not associated with different infant feeding cultures. Most likely they are associated with the higher proportion of low-income and low-education mothers in the southern regions of Italy. These disparities should be taken into account in the formulation of breastfeeding programmes to avoid increasing, and possibly to decrease, the current gaps.

## Competing interests

The author(s) declare that they have no competing interests.

## Authors' contributions

AC and SQR planned and designed the study. SQR and RB carried out the field work, performed and processed all the interviews, and helped with the qualitative analysis. CB, RP and AC carried out the statistical analysis and interpretation. All the authors contributed equally to writing the paper.
